# Unexpected low soil-transmitted helminth prevalence in the Butha-Buthe district in Lesotho, results from a cross-sectional survey

**DOI:** 10.1186/s13071-017-1995-x

**Published:** 2017-02-08

**Authors:** Wendelin Moser, Niklaus Daniel Labhardt, Molisana Cheleboi, Josephine Muhairwe, Jennifer Keiser

**Affiliations:** 1 Department of Medical Parasitology and Infection Biology, Swiss Tropical and Public Health Institute, Basel, Switzerland; 2 University of Basel, Basel, Switzerland; 3 Department of Medical Services and Diagnostic, Swiss Tropical and Public Health Institute, Basel, Switzerland; 4 Laboratory Services, Seboche Hospital, Butha-Buthe, Lesotho; 5 SolidarMed, SolidarMed Lesotho, Butha-Buthe, Lesotho

**Keywords:** Soil-transmitted helminths, Neglected tropical diseases, *Ascaris lumbricoides*, Hookworm, *Trichuris trichiura*, Butha-Buthe, Lesotho

## Abstract

**Background:**

Soil-transmitted helminth (STH) infections with *Ascaris lumbricoides*, hookworm and *Trichuris trichiura* affect large parts of the world’s population. For the implementation of national STH control programs, e.g. preventive chemotherapy (treatment with albendazole and mebendazole), the spatial distribution and prevalence of STH infections must be known. However, for Lesotho only little data were available and the STH distribution remains largely unknown.

**Methods:**

In early 2016, a cross-sectional parasitological STH survey was conducted including six different primary schools in the Butha-Buthe district of Lesotho. In each school stool samples were collected from 50 children (age 8–14 years) and analysed with a duplicate Kato-Katz thick smear for the presence of *A. lumbricoides*, hookworm and *T. trichiura*.

**Results:**

A total of 301 children provided a stool sample. All children were negative for *A. lumbricoides* and *T. trichiura*. Only two children from one primary school showed a light hookworm infection.

**Conclusion:**

Our data indicate a low prevalence of STH infections in the Butha-Buthe district of Lesotho. Additional parasitological surveys on the prevalence and the spatial distributions of STH infections across the entire country of Lesotho are needed.

## Background

Neglected tropical diseases (NTDs) are a group of communicable diseases, which mainly affect poor, rural communities in tropical and sub-tropical regions around the world. Soil-transmitted helminthiasis is an important NTD caused by infections with the nematodes *Ascaris lumbricoides*, hookworm (*Necator americanus* and *Ancylostoma duodenale*) and *Trichuris trichiura*. Around the globe, about 5.3 billion people are living in an area where soil-transmitted helminth (STH) transmission occurs. Approximately 1.5 billion people are infected with at least one of the nematodes, with highest infection numbers for *A. lumbricoides* (820 million), followed by *T. trichiura* (470 million) and hookworm (420 million) [1, 2].

A chronic STH infection causes numerous health conditions including dietary deficiency, physical delayed cognitive development and iron deficiency [3–6]. STH infections are responsible for an estimated burden of 3.4 million disability-adjusted life years (DALYs) [7]. Preventive chemotherapy with repeated administration of albendazole and mebendazole is the current strategy of choice to reduce moderate and heavy STH infections in pre-and school-aged children [8, 9]. Preventive chemotherapy is promoted by the World Health Organization (WHO), due to its fast and strong impact on morbidity and simple implementation [10].

In order to guide STH control programs (e.g. preventive chemotherapy), parasitological and epidemiological data are required. In more detail, data on the district and national prevalence and the intensities of infections [11] are necessary to initiate a locally adapted control program. Given that the main public health problems of Lesotho are HIV/AIDS and tuberculosis [12] data on STHs are very scarce. Neither published prevalence data nor maps of Lesotho presenting the spatial and temporal distribution of helminth infections are available [2, 13, 14]. In the WHO NTD country profile for Lesotho of the year 2010 STHs are listed as endemic and approximately half a million pre-and school-aged children are estimated to be in need of preventive chemotherapy. In 2010, about 150,000 pre-school aged children were treated with albendazole or mebendazole. Since 2010, the estimated number of pre- and school-aged children requiring preventive chemotherapy remained unchanged, however the number of tablets administered were not recorded [15, 16].

In early 2015, the Ministry of Health of Lesotho conducted a country wide survey for mapping NTDs [17]. Five schools per district including 50 children per school were examined for STH infections. STH infections were observed to be a significant public health problem for the Butha-Buthe district with highest prevalence for *T. trichiura* (unpublished observations). The aim of our study was to confirm these results generated by the Ministry of Health of Lesotho in the Butha-Buthe district and to present the first time, openly available knowledge on STH prevalence in Lesotho.

## Methods

### Study area and procedures

**Fig. 1:**
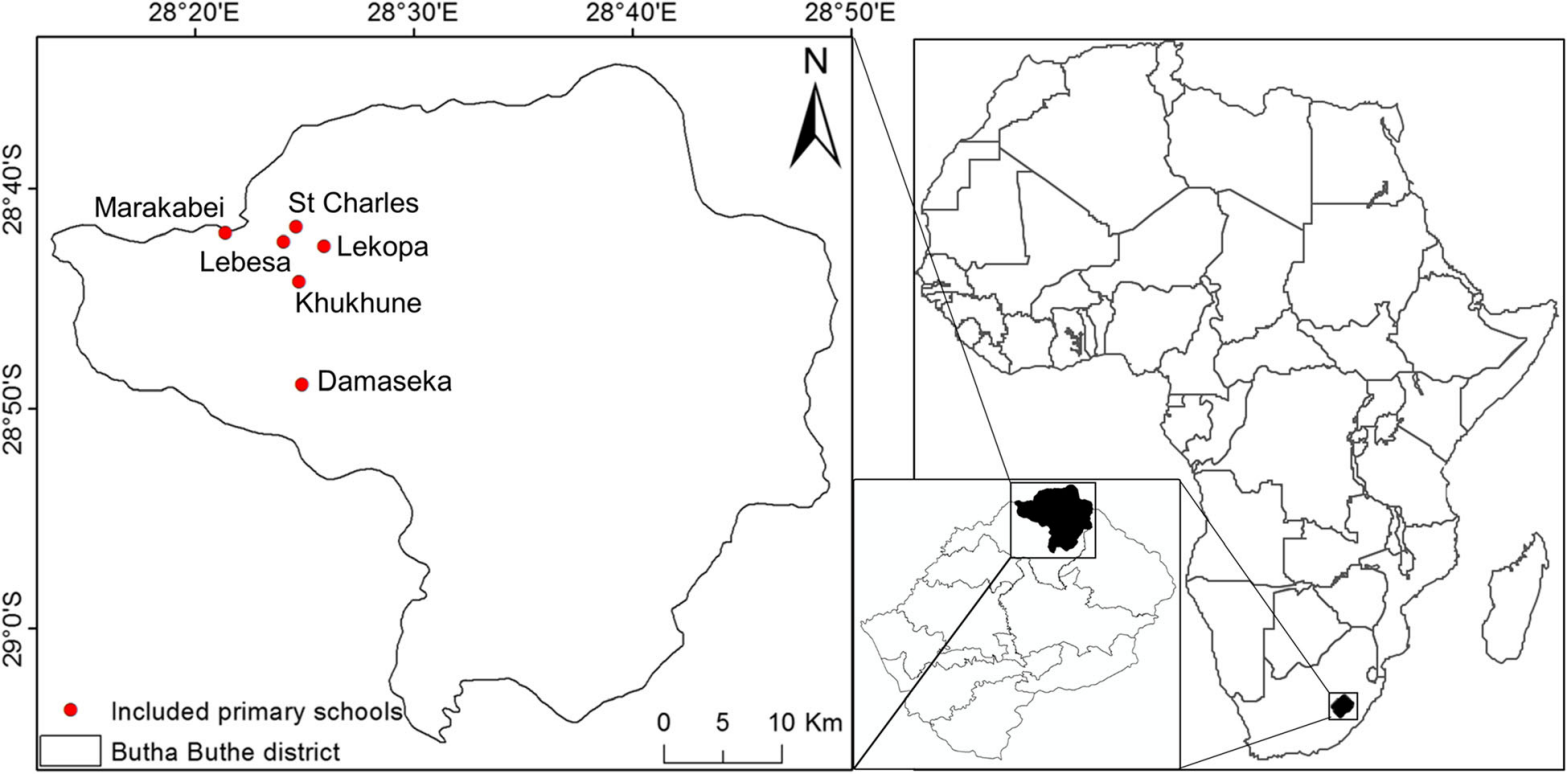
Study area. Map of the six included primary schools of the Butha-Buthe district in Lesotho

This cross-sectional study was conducted from 25 April until 6 May 2016 in Butha-Buthe, the most northern district in Lesotho (Fig. 1). The population of the district slightly exceeds 100,000 inhabitants (Lesotho Bureau of Statistics), of which about 30,000 live in the district’s only city, while the remaining inhabitants live in rural, partially very remote mountainous areas. The study was designed based on WHO guidelines for managing helminth control in school-aged children [11]. We aimed to include 50 children aged 8 to 14 from five randomly chosen primary schools in the Seboche catchment area (Marakabei, St. Charles, Lekopa, Lebesa and Khukhune, Fig. 1) and one primary school from the Linakeng catchment area (Damaseka).

On the collection day stool containers labelled with a unique identification number were distributed randomly to 52 eligible children, who were asked to provide a stool on the same morning. An additional two children were invited to participate in the study (total = 52) to account for children who were not able to provide a stool on the same morning. All containers from one school per day were collected and transferred to the laboratory of the St. Charles Seboche Mission Hospital. From each stool sample duplicate Kato-Katz thick smears (41.7 mg each) were prepared [18] and read within 1 h after preparation to avoid overclearing of hookworm eggs [19]. The slides were analysed under a light microscope for *A. lumbricoides*, hookworm and *T. trichiura* eggs by three experienced technicians. Subsequently, an independent quality control of sample results (approximately 10%) was conducted. The quality control tolerance margin was chosen according to the suggestion of Speich et al. [20] and in case of discrepancies, the exceeding slides were re-read and discussed until consensus was reached.

An infection was classified as positive if at least one parasite egg in at least one the two Kato-Katz thick smears was present. The eggs counted in both slides were added, divided by two and multiplied by a factor of 24 to obtain egg per gram (epg) of stool values. Infection intensities were stratified into light, moderate and heavy according to WHO cut-offs [21]. The prevalence was calculated as percentage of positive children. All data were analysed using the statistical program STATA Version 14 (Stata Corp. LP; Texas, USA).

## Results

A total of 312 children were invited to participate in the study. Of these, 301 children returned a stool sample, while 11 were not able to defecate on the same morning. The aim of collecting 50 samples per school was achieved, except for Khukhune primary school (*n* = 49), where 3 children were not able to provide a stool sample. Fifty samples were collected in the primary schools of St. Charles, Lebesa, Marakabei and Damaseka and 52 in Lekope (Table 1).

**Table 1:** Characteristics and infections numbers of the included children from the primary schools Marakabei, St. Charles, Lekopa, Lebesa, Khukhune and Damaseka of the Butha-Buthe district in Lesotho

School	St. Charles	Lebesa	Marakabei	Lekopa	Khukhune	Damaseka	Total
Invited children (*n*)	52	52	52	52	52	52	312
Participating children (*n*)	50	50	50	52	49	50	301
Age (yrs) average (± SD)	10.4 ± 1.6	10.5 ± 1.9	11.0 ± 1.7	9.3 ± 1.0	11.0 ± 1.3	11.4 ± 1.6	10.6 (±1.7)
Boys, *n* (%)	28 (56.0)	26 (52.0)	20 (40.0)	25 (48.1)	22 (44.9)	18 (36.0)	139 (46.2)
Grade, *n* (%)							
3	12 (24.0)	11 (22.0)	0 (0)	16 (30.8)	0 (0)	8 (16.0)	47 (15.6)
4	15 (30.0)	11 (22.0)	18 (36.0)	22 (42.3)	10 (20.4)	4 (8.8)	80 (26.6)
5	8 (16.0)	9 (18.0)	11 (22.0)	14 (26.9)	14 (28.6)	13 (26.0)	69 (22.9)
6	15 (30.0)	16 (32.0)	14 (28.0)	0 (0)	16 (32.7)	19 (38.0)	80 (26.6)
7	0 (0)	3 (6.0)	7 (14.0)	0 (0)	9 (18.4)	6 (12.0)	25 (8.3)
Infected children, *n* (%)							
*Ascaris lumbricoides*	0 (0)	0 (0)	0 (0)	0 (0)	0 (0.0)	0 (0)	0 (0)
Hookworm	0 (0)	0 (0)	0 (0)	0 (0)	0 (0.0)	2 (4.0)	2 (0.7)
*Trichuris trichiura*	0 (0)	0 (0)	0 (0)	0 (0)	0 (0.0)	0 (0)	0 (0)

The age of included children ranged from 8 to 14 with an average of 10.6 years (Table 1). Slightly more girls (53.8%) were included in the study. The vast majority of participants were visiting grade 4 to 6 (76.1%) with only few children registered in grades 3 and 7 (23.9%).

All studied children from the schools St. Charles, Lebesa, Marakabei, Lekope and Khukhune were negative for *A. lumbricoides*, hookworm and *T. trichiura* (Table 1). From 50 children examined in the Damaseka primary school, two showed a light (epg < 300) hookworm infection (4.0%) and none were infected with *A. lumbricoides* and *T. trichiura*.

## Discussion

The most recent estimates from 2010 reported approximately 1.5 billion STH-infected people, with highest infection numbers found in Asia, followed by sub-Saharan Africa [2]. In 2014 the global coverage of preventive chemotherapy with anthelmintic drugs (albendazole and mebendazole) reached 47% [22]. To further increase the treatment coverage and adequately plan and implement STH control programs, knowledge about the spatial distribution and helminth species-specific prevalences are necessary. Despite an ongoing global effort to map the abundance of STH, there are no publicly available data for Lesotho [2, 13, 14]. Official estimates hint that soil-transmitted helminthiasis is a significant public health burden in Lesotho. In more detail, the very first estimate retrieved from the WHO preventive chemotherapy and transmission (PCT) databank, reports a number of 750,000 pre- and school-aged children requiring anthelmintic drugs in Lesotho and dates back to the year 2003 [15]. For the year 2015, the estimates still suggested a high number (520,000) of pre- and school-aged children in need of preventive chemotherapy for soil-transmitted helminthiasis. In addition, an unpublished survey conducted by the Ministry of health in Lesotho in early 2015 revealed moderate prevalence rates for *A. lumbricoides* and *T. trichiura* infections in the whole of Lesotho. Published data are not available and the exact number of STH-infected children, requiring preventive chemotherapy, remains unknown. We aimed to fill this knowledge gap and present for the first time findings on the prevalence of STHs in Lesotho, specifically in the Butha-Buthe district. According to the above mentioned unpublished survey conducted by the Ministry of Health of Lesotho Butha-Buthe revealed highest prevalence among all districts in Lesotho.

Our study showed no STH infections in school-aged children attending the five primary schools of the Seboche catchment area. Evaluating STH infections in only one catchment area is clearly a limitation of our study. However, in order to verify our negative results, we added one primary school (Damaseka) of another catchment area. The primary school of Damaseka was already involved in the previous, unpublished Ministry of Health survey and revealed high *T. trichiura* prevalence. Despite even the use of a more sensitive diagnostic method compared to the previous NTD survey (duplicated Kato-Katz *vs* direct microscopy) [23, 24] and a quality control, we could not confirm the high *T. trichiura* prevalence, while two children were infected with hookworm. Retrospectively, for settings with such a low prevalence, diagnostic methods with higher sensitivity would have been required, as e.g. a multiple Kato-Katz or polymerase chain reaction (PCR) [24, 25].

Our results are in contrast to the high prevalence reported by the NTD-survey for the Butha-Buthe district conducted in nearby schools and the following points are offered for discussion. First, the prevalence of STHs is highly variable even in small areas and influenced by the difference of locally restricted factors, e.g. geological composition and soil types [26, 27]. Since the Seboche catchment area was not involved in the previous NTD-survey, the difference between the moderate prevalence reported earlier and no infection observed in our study could be attributed to spatial distribution.

Secondly, effective sanitation is in place in the study area. In more detail, during collection of the stool samples in the primary schools information on the availability of sanitation at the school and village level in the Seboche catchment area was obtained. Each of the primary schools of the Seboche catchment area had a clean, well-functioning toilet and all children were encouraged by the teacher to use the toilets, while open defecation is prohibited. Teachers and local collaborators reported that the vast majority of homes around the school are equipped with an adequately working toilet (commonly ventilated pit latrines). Of note, this is not the case in all schools and villages of Lesotho and the Butha-Buthe district. To overcome this problem one large project financed by the Millennium Challenge Cooperation (MCC, USA) currently conducts health trainings, the construction of rural water systems to provide safe drinking water and the construction of ventilated pit latrines in order to improve the health situation in the least developed villages [28, 29], including some adjacent villages of Damaseka (personal observation).

Third, the history of treatment might be responsible for the differing prevalence rates observed by us and the Ministry of Health of Lesotho. However, to our knowledge primarily pre-schoolers are treated in Lesotho. In more detail, to tackle the problem of STH infections in Lesotho, the national guidelines recommend the administration of albendazole or mebendazole twice a year to pre-school aged children (from the age 1 to 5) [30, 31]. Data from the WHO PCT databank report solely the treatment of pre-school aged children in the year 2007 and 2010, while school-aged children were not included in these programs [15]. In subsequent years, albendazole was offered free of charge to pre-school aged children at the health centre level (personal communication). The extend of this deworming program was highly depended on the parents’ motivation to get their children treated.

## Conclusions

A NTD-survey from 2015 indicated moderate STH prevalence for the Butha-Buthe district, however we could not confirm these findings. Based on our data, STH infections are not a public health problem for the area of Seboche and only marginally for the Linakeng catchment area. It is worth highlighting that we only investigated a handful of schools, which does not allow drawing a general conclusion for the situation for the entire district. To generate further data on the spatial distribution of STH in Butha-Buthe, a random sampling of the entire district is necessary. Subsequently, a detailed national survey should be conducted, which allows revising the national deworming policy.
